# Saline is as effective as nitrogen scavengers for treatment of hyperammonemia

**DOI:** 10.1038/s41598-017-12686-9

**Published:** 2017-10-13

**Authors:** G. van Straten, M. G. M. de Sain-van der Velden, I. M. van Geijlswijk, R. P. Favier, S. J. Mesu, N. E. Holwerda-Loof, M. van der Ham, H. Fieten, J. Rothuizen, B. Spee, N. M. Verhoeven-Duif

**Affiliations:** 1 Department of Clinical Sciences of Companion Animals, Faculty of Veterinary Medicine, Utrecht University, Utrecht, The Netherlands; 2 Department of Medical Genetics, Wilhelmina Children’s Hospital, University Medical Centre (UMC), Utrecht, The Netherlands; 3 Pharmacy Department, Faculty of Veterinary Medicine, Utrecht University, Utrecht, The Netherlands

## Abstract

Urea cycle enzyme deficiency (UCED) patients with hyperammonemia are treated with sodium benzoate (SB) and sodium phenylacetate (SPA) to induce alternative pathways of nitrogen excretion. The suggested guidelines supporting their use in the management of hyperammonemia are primarily based on non-analytic studies such as case reports and case series. Canine congenital portosystemic shunting (CPSS) is a naturally occurring model for hyperammonemia. Here, we performed cross-over, randomized, placebo-controlled studies in healthy dogs to assess safety and pharmacokinetics of SB and SPA (phase I). As follow-up safety and efficacy of SB was evaluated in CPSS-dogs with hyperammonemia (phase II). Pharmacokinetics of SB and SPA were comparable to those reported in humans. Treatment with SB and SPA was safe and both nitrogen scavengers were converted into their respective metabolites hippuric acid and phenylacetylglutamine or phenylacetylglycine, with a preference for phenylacetylglycine. In CPSS-dogs, treatment with SB resulted in the same effect on plasma ammonia as the control treatment (i.e. saline infusion) suggesting that the decrease is a result of volume expansion and/or forced diuresis rather than increased production of nitrogenous waste. Consequentially, treatment of hyperammonemia justifies additional/placebo-controlled trials in human medicine.

## Introduction

Hyperammonemia is a dangerous condition that can lead to hepatic encephalopathy (HE) and in severe cases to coma and death^1–3^. Detoxification of ammonia occurs mainly through conversion into urea via the liver-specific urea cycle or by conversion to glutamine through glutamine synthetase^4–6^. Hyperammonemia occurs frequently in cases of impaired endogenous hepatic detoxification (e.g. acute liver failure and liver cirrhosis), when the portal circulation bypasses the liver (congenital or acquired portosystemic shunts, CPSS and APSS respectively) and in a variety of metabolic conditions such as urea cycle enzyme deficiencies (UCED).

**Figure 1:**
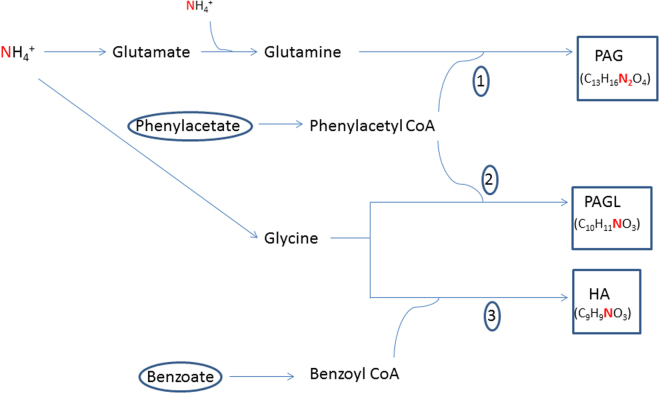
Schematic overview of benzoate and phenylacetate metabolism and the urea cycle. Phenylacetate conjugates with glutamine by glutamine N-phenylacetyltransferase (reaction 1) or glycine by glycine N-phenylacetyltransferase (reaction 2) to form phenylacetylglutamine (PAG) or phenylacetylglycine (PAGL) respectively. Benzoate conjugates with glycine by glycine N-benzoyltransferase (reaction 3) to form hippuric acid (HA). Treatment with sodium phenylacetate and sodium benzoate may act as ‘scavengers’ for surplus ammonium that is not being converted to urea in the urea cycle.

Treatments of acute hyperammonemia aim at a reduction of ammonia formation and a rapid excretion of as much waste nitrogen as possible. Since the 1980s, severe hyperammonemia in patients with UCED has been treated with sodium benzoate (SB) and/or sodium phenylacetate (SPA). These substances were reported to lower ammonia concentration by activating alternative pathways of nitrogen excretion and thus, scavenge excess nitrogen for disposal^7–11^. Benzoate conjugates with glycine (catalysed by glycine N-benzoyltransferase) to form hippuric acid (HA), and phenylacetate conjugates with glutamine (catalysed by glutamine N-phenylacetyl transferase) to form phenylacetylglutamine (PAG) (Fig. 1). The latter is reported to occur only in humans and higher primates. In other species, phenylacetate conjugates with glycine to form phenylacetylglycine (PAGL)^12,13^, (Fig. 1). HA, PAGL and PAG are excreted by the kidneys removing one (HA and PAGL) and two (PAG) moles of waste nitrogen for each mole of benzoate and phenylacetate, respectively^14,15^, (Fig. 1).

Although both SB and SPA have been used therapeutically for many years, we still lack a high level of evidence (such as systematic reviews of randomized controlled trials (RCTs)) supporting the efficacy of these treatments in humans and animals. Consequently, consensus statements and suggested guidelines^16–18^ supporting the use of one or both substances in the management of acute hyperammonemia are primarily based on non-analytic studies such as case reports and case series. Additionally, ammonia concentration did not decrease in all studied patients and in some patients, ammonia levels even increased^7,10,11,19^. In mice, treatment with SB increased ammonia concentrations leading to increased mortality^20–23^ and in rats, SB was ineffective despite the significant excretion of urinary HA^24^.

One of the main reasons for the lack of large scale placebo-controlled trials in UCED patients is that we cannot ethically withhold the only possible effective treatment. On the other hand, from a medical and economic stand point, it is of utmost importance that such studies would be conducted in order to provide the strongest evidence of the effectiveness of a treatment. For this reason, a large animal model is required. In dogs, UCED are extremely rare^25,26^ and CPSS is the most common cause of hyperammonemia and HE^27,28^. CPSS result in severe reduction of ureagenesis, reduced expressions of urea cycle enzymes and consequently hyperammonemia^29,30^. Clinical symptoms of hyperammonemia in dogs (e.g. depression, ataxia, convulsions and coma) are highly similar to those seen in humans.

As previously acknowledged, CPSS in dogs is used as naturally occurring model for human HE^31^. This dog model allows, for the first time, the use of a placebo control. In human treatment protocols SB and SPA are always intravenously administered in a 10% dextrose solution. To avoid some effects of clearance of ammonia due to this hypertonic infusion, we chose saline 0.9% in equivalent volume for the drug administration and as placebo. In this study, we use healthy dogs and dogs with CPSS to evaluate safety and efficacy of treatment with SB and SPA for acute hyperammonemia. The aims of this study were to assess the safety, pharmacokinetics and metabolism of SB and SPA in healthy dogs (phase I study) and to evaluate the efficacy of these treatments for reduction of hyperammonemia in dogs with CPSS (phase II study).

## Materials and Methods

### Study design

**Figure 2:**
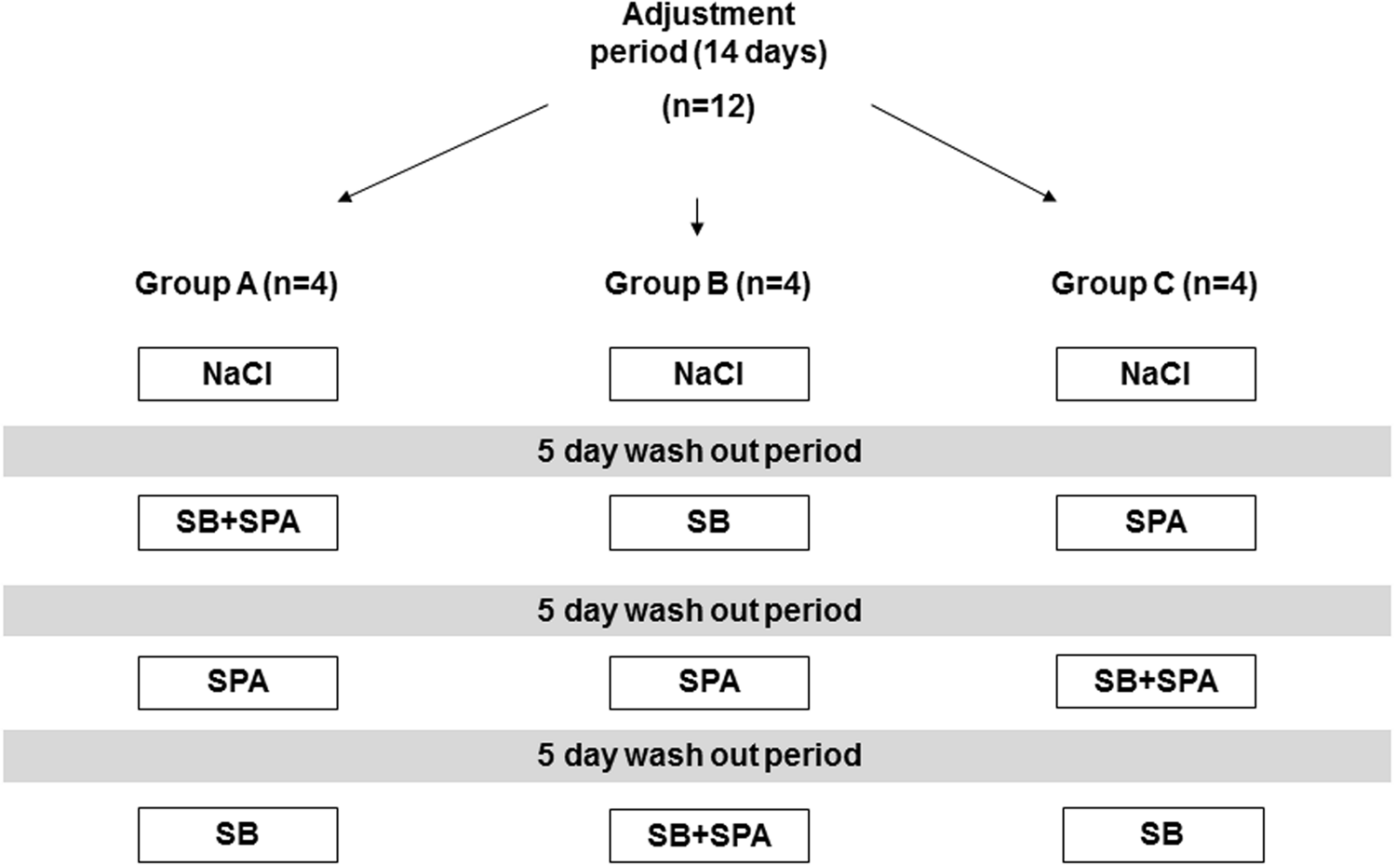
Schematic overview of the Latin square design. Twelve healthy dogs were evenly divided into three groups. The first treatment was NaCl 0.9% (NaCl) in all groups. Sodium benzoate (SB), sodium phenylacetate (SPA) and the combination of both drugs (SB + SPA) were then administered to each group in a different sequence to prevent a possible sequence effect. A wash out period of minimal five days was obtained between each treatment in order to avoid carry-over effects. The substances were administered via a central venous catheter in the jugular vein that was placed before each treatment. Substances were given as a loading dose infusion over a period of 120 minutes.

Phase I was a placebo controlled, cross-over, open-label study designed to assess safety, tolerability, pharmacokinetics and efficacy of SB and SPA in monotherapy and in combination. Twelve healthy dogs were randomly allocated to three groups each consisting two males and two females. According to a Latin-square design (Fig. 2), the dogs were infused with sodium benzoate (SB, 250 mg/kg), sodium phenylacetate (SPA, 250 mg/kg), SB + SPA (250 mg/kg of each substance) and NaCl 0.9% (control) in an equivalent volume given with SB or SPA. Substances were given as a loading dose infusion, via a jugular catheter, over a period of 120 minutes. The doses and administered volumes were equivalent to those recommended in human treatment protocols^16,17^. After phase I, phase II studied, safety, tolerability, pharmacokinetics and efficacy of SB in CPSS dogs. In all dogs, loading dose and administration routes were identical to those described in phase I. Treatment started with NaCl 0.9% and after a wash out period of 24 hours SB (250 mg/kg) was administered. All procedures were approved by and performed according to the standards of the Ethical Committee of Animal Experimentation of Utrecht University under application number 2011.III.12.119. All experiments were performed in accordance with relevant guidelines and regulations. For the client-owned dogs a mandatory written consent from the patient owners was also obtained.

### Animals

For phase I, 12 healthy dogs, aged two to six years, were used. All dogs belonged to the Department of Clinical Sciences of Companion Animals at Utrecht University, The Netherlands. Seven beagles (five males and two females) weighing 11.0–19.5 kg and five mixed breed dogs (one male and four females) weighing 10.0–12.4 kg were included. During the experiments, the dogs were housed in individual metabolic cages and were fed a protein rich diet (24 gram crude protein per 100 gram dry matter) once daily. Dogs were fed two hours before treatment to evaluate possible effects on the postprandial ammonia peak concentrations that occur six hours after feeding^32^. Treatment effects on ammonia concentrations in healthy dogs were expected at Cmax (end of the bolus infusion) and the time of the peak ammonia concentration. In the wash out periods, the dogs were housed in pairs in indoor-outdoor runs, fed the protein rich diet once daily and water was available ad libitum.

All dogs were accustomed to the diet during the two weeks prior to the experiment. Phase II involved four dogs with CPSS. Dog one was a mixed breed, three-year old female with an intrahepatic shunt (IHPSS). Dog two was a six-month old Cairn terrier, intact male with an extrahepatic shunt (EHPSS). Dog three was a four-month old Nova Scotia Duck tolling retriever, intact male with an IHPSS and dog four was a one year and three months-old Shih Tzu intact female with an EHPSS. All dogs presented with one or more HE symptoms (lethargy, disorientation, ataxia, blindness, and periods of unconsciousness), and hyperammonemia (reference interval 15–45 µmol/l). Additional symptoms reported in dog four were vomiting and anorexia that started three days prior to referral. Dog one belonged to the Department of Clinical Sciences of Companion Animals; all other dogs were client-owned. CPSS was diagnosed in all four dogs according to previously described methods^33^ All four dogs were fed a standardized commercial diet (Royal Canine, Hepatic®) before and during the study, and no medication was given to any of the dogs two weeks prior to the study.

### Drug preparation

For phase I and II, an injectable formulation of sodium benzoate (SB) (Spruyt Hillen BV, IJsselstein, Netherlands) and sodium phenylacetate (SPA) (Sigma-Aldrich, Steinheim, Germany) were prepared by the Pharmacy Department of the Faculty of Veterinary Medicine, Utrecht University. The compounded infusion solutions contained SB (10 mg/ml), SPA (10 mg/ml), or SB + SPA (10 mg/ml of each substance). The solutions were filtered through a 0.2 µm filter and filled per 500 ml in infusion bottles. The bottles were sterilized at 121 °C for 15 minutes. Acidity of the solutions for infusion was: SB, pH = 7.5; SPA, pH = 6.8; SB + SPA, pH = 7.1. CPSS dogs (phase II) are predisposed to hypoglycemia due to reduced liver function. Therefore, glucose was added to both the SB as the saline treatment (solution 1%) in order to minimize gluconeogenesis and consequently extra ammonia production.

### Blood and urine collection

Blood samples for the quantification of the active substance of SB (benzoic acid, BA); the active substance of SPA (phenylacetic acid, PAA); and metabolites, phenylacetylglutamine (PAG), phenylacetylglycine (PAGL), and hippuric acid (HA), were collected by jugular venipuncture just before treatment (T) 0 and at 2, 3, 4, 6, 8 and 24 hours after starting the treatment. For the phase II study, blood was additionally sampled at 12 and 18 hours after treatment initiation. In addition, a complete blood count (CBC), biochemical profile, ammonium concentration and pH measurements were also performed at all the above-mentioned time points. Blood was immediately transferred to ice-chilled sodium-ethylenediaminetetraacetic acid (Na-EDTA) coated tubes (ammonia analysis) or heparin-coated tubes (BA, PAA, PAG, PAGL and HA analyses). Heparinized blood samples were centrifuged at 4 °C and plasma was collected and stored at −20°C until assayed. Urine samples were collected via a urine catheter for measurements of BA, PAA, PAGL, PAG and HA. Urine analysis was performed prior to treatment (T0) and at 3, 6, 12, 18 and 24 hours after treatment initiation. The urine catheter was placed before each treatment and was removed directly after the last urine collection (T24). Recovery of BA as a percentage HA was calculated from the mean of total urine HA excreted (micromole) divided by the mean total SB administrated (micromole). Recovery of PAA as percentage PAGL or PAG was calculated from the mean of total urine PAGL and PAG excreted (micromoles) divided by the mean total administered SPA (micromoles). Calculation of the total urine nitrogen excretion was based on the fact that HA and PAGL are both products of glycine conjugation and therefore contain one nitrogen molecule each. PAG is a product of glutamine conjugation and contains two nitrogen molecules (Fig. 1).

### Analytical methods, reagents

Benzoic acid (BA), hippuric acid (HA), 4-phenylbutyric acid (PBA), PAA and methanol were obtained from Sigma-Aldrich (Steinheim, Germany); phenylacetylglycine (PAGL) and phenylacetylglutamine (PAG) were purchased from Bachem (Bubendorf, Switzerland); Benzoic acid (Ring-D5, 98% (BA-D5)) were purchased from Cambridge Isotopes (Andover, USA); N-Benzoyl-D5-glycine (99.2% (HA-D5)), Nα-(Phenyl-D5-acetyl)-L-glutamine (99.1% (PAG-D5)), Phenyl-D5-acetic acid (98.5% (PAA-D5)) and 4-Phenylbutyric-D11 acid (99.4% (PBA-D11)) from CDN Isotopes (Quebec, Canada). Formic acid and ethanol were purchased from Merck (Darmstadt, Germany). Acetonitrile was obtained from Biosolve (Valkenswaard, The Netherlands).

### Analytical methods, standard solutions and calibration curve

Stock solutions (mmol/l): BA, 8.5; HA, 5.4; PBA, 6.0; PAGL, 5.2; PAG, 3.9; PAA, 7.3, were prepared in 70% ethanol. A 10-point calibration curve was prepared by diluting these stock solutions in methanol, with final concentrations (µmol/l): BA, 17.0; HA, 108.0; PBA, 119.4; PAGL, 104.4; PAG, 78.2; PAA, 146. Stock solutions (1 mg/ml) of the stable isotope D-labeled compounds (BA-D5, HA-D5, PBA-D11, PAG-D5 and PAA-D5) were prepared in methanol. An internal standard working solution was prepared by diluting these stock solutions in a mixture (10 ng/ml) with methanol.

### Analytical methods, quality control samples (QC’s)

For validation of the method in urine and for quality control, three different urine samples were used. These samples were also analysed with each batch of urine samples. For validation of the method in plasma and for quality control, human heparinized plasma was pooled and spiked at three different levels with the compounds of interest. These samples were included in each batch of plasma samples.

### Analytical methods, instruments

A Quattro Ultima Platinum triple quadrupole mass spectrometer (tandem MS) (Waters, Manchester, UK) interfaced with an electron spray ionization source and equipped with an Alliance 2795 HPLC (Waters, Etten-Leur, The Netherlands) was used. Masslynx software (Version 4.0, SP 4, Waters, Manchester, UK) was used for instrument control, data acquisition and data processing.

### Analytical methods, chromatographic and mass spectrometric conditions

Chromatographic and mass spectrometric conditions on the HPLC/MS/MS system were used as described before^34^ with some slight modifications. In short, an extra component, phenylacetylglycine, was added to the method (MRM transition m/z 192.1 > 74.1, cone voltage 45 V and collision energy 8 eV). For chromatographic separation, acetonitril was used instead of methanol, and a gradient was used to flush the column in each run. Initial conditions were 100% solvent A (0.01% formic acid/acetonitril 35/65, v/v) for 2.5 minutes. In 0.1 minute, the system switched to 100% acetonitrile for 1.05 minutes. In 0.15 minutes, the system switched back to the initial conditions and a total run time of 6 minutes was used to equilibrate the column.

### Sample preparation

Sample preparation procedure was performed as described in detail elsewhere^34^. Urine samples and plasma samples were diluted with, respectively, MilliQ and 0.9% sodium chloride to achieve concentrations of the compounds that lay within the range of the calibration curve.

### Complete Blood Count (CBC), biochemical profile, ammonium concentration and pH

Packed cell volume, and total and differential leukocyte counts were measured using the ADVIA® 2120i System (Siemens Healthcare Diagnostics BV, Den Haag, The Netherlands). Sodium, potassium, calcium, albumin, creatinine, BUN, alanine aminotransferase (ALAT), alkaline phosphatase (AP) and bile acids (BA) were determined with the UniCel DxC 600® assay (Beckman Coulter Nederland BV, Woerden, The Netherlands). Blood pH was measured using the Rapidlab® 1265 (Siemens Healthcare Diagnostics BV, Den Haag, The Netherlands). Ammonia concentrations were measured within 10 minutes after collection, using a micro diffusion method, PocketChem™ BA (Menarini Benelux BV, Valkenswaard, The Netherlands). In cases where ammonia concentrations exceeded the measurable value (286 µmol/l), samples were diluted as previously described^35^.

### Pharmacokinetic analysis

BA and PAA clearance was considered log linear and fitted all data into an initially two-compartment first-order pharmacokinetic analysis model. Pharmacokinetic calculations of the elimination half-life (T½), volume of distribution (Vd), maximum concentration (Cmax), and area under the concentration versus time curve (AUC) were performed using MwPharm software version 3.15 (Mediware, Heerenveen, The Netherlands).

### Statistics

Biochemical and hematological blood parameters in the phase I study were investigated by mixed model analysis in which blood parameters were dependent variables and time (as a factor), treatment (NaCl 0.9% as reference category, SB, SPA and SB + SPA), and order of treatment from the Latin square design, were modelled as fixed factors. Validity of the model was checked by inspecting QQ-plots and by plotting residuals against fitted values and by inspecting residuals per animal. For sodium, potassium, calcium, albumin and pH, time points 0, 2, 3, 4, 6, 8 and 24 hours after treatment were available. For BUN, creatinine, alkaline phosphatase, ALT, total protein, Ht and leukocytes, time points 0 and 24 post treatment were available.

Differences in pharmacokinetic analysis: After performing the F-test to check variances, the T-test Unequal Variances Assumed was applied to compare T½, AUC and Cmax of both active substances alone and in combination for phase I (Excel Microsoft Office Professional Plus 2013). Phase II results for T½ and AUC after SB treatment were analysed for comparability with phase I results. Differences in HA, PAG and PAGL in blood between SB, SPA and SB + SPA treatment were investigated by a Wilcoxon-signed rank test with continuity correction at each time point (0, 2, 3, 4, 6, 8, and 24 hours after treatment). In urine, differences in HA between SB and SB + SPA treatment and in PAG and PAGL between SPA and SB + SPA treatment were investigated by a Wilcoxon-signed rank test with continuity correction at each time point (0, 3, 6, 12, 18, and 24 hours post treatment). Different levels of ammonia in the blood between NaCl 0.9% and SB; NaCl 0.9% and SPA; and NaCl 0.9% and SB + SPA were investigated by a Wilcoxon-signed rank test with continuity correction at each time point (0, 2, 3, 4, 6, 8, and 24 hours after treatment).

### Data availability

All data generated or analysed during this study are included in this published article (and its Supplementary Information files).

## Results

### Safety and tolerability

Eleven dogs in the phase I trial completed all treatments (NaCl 0.9%, SB, SPA and SB + SPA). One dog developed a pyometra three weeks after the start of the trial and did not participate in the last treatment (SB administration). The only adverse events that were seen were vomiting and lethargy. Two dogs vomited during SB + SPA administration, one dog during SB administration and one during administration of NaCl 0.9%. Lethargy was seen in the same dogs that vomited. Both adverse events were temporary and resolved without specific treatment shortly after administration of the drugs. No significant changes were found in plasma concentrations of alanine aminotransferase (ALAT), alkaline phosphatase (AP), and creatinine 24 hours after treatment initiation (T24). ALAT and AP levels at the end of the study period were significantly higher than those measured prior to the first treatment. However, multivariate analysis has shown that the increase in both enzymes was not related to treatment. Hematocrit (Ht), bile acids and urea (BUN) plasma concentrations were significantly lower and leucocytes were significantly higher at T24. Treatments did not influence the plasma concentrations of albumin, calcium, sodium and potassium but pH levels were significantly increased after SB, SPA and SB + SPA in comparison to NaCl 0.9% administration. Despite the increase in pH, none of the dogs developed symptoms related to metabolic alkalosis. Results are summarized in Supplementary Table S1.

### Pharmacokinetic analysis

**Figure 3:**
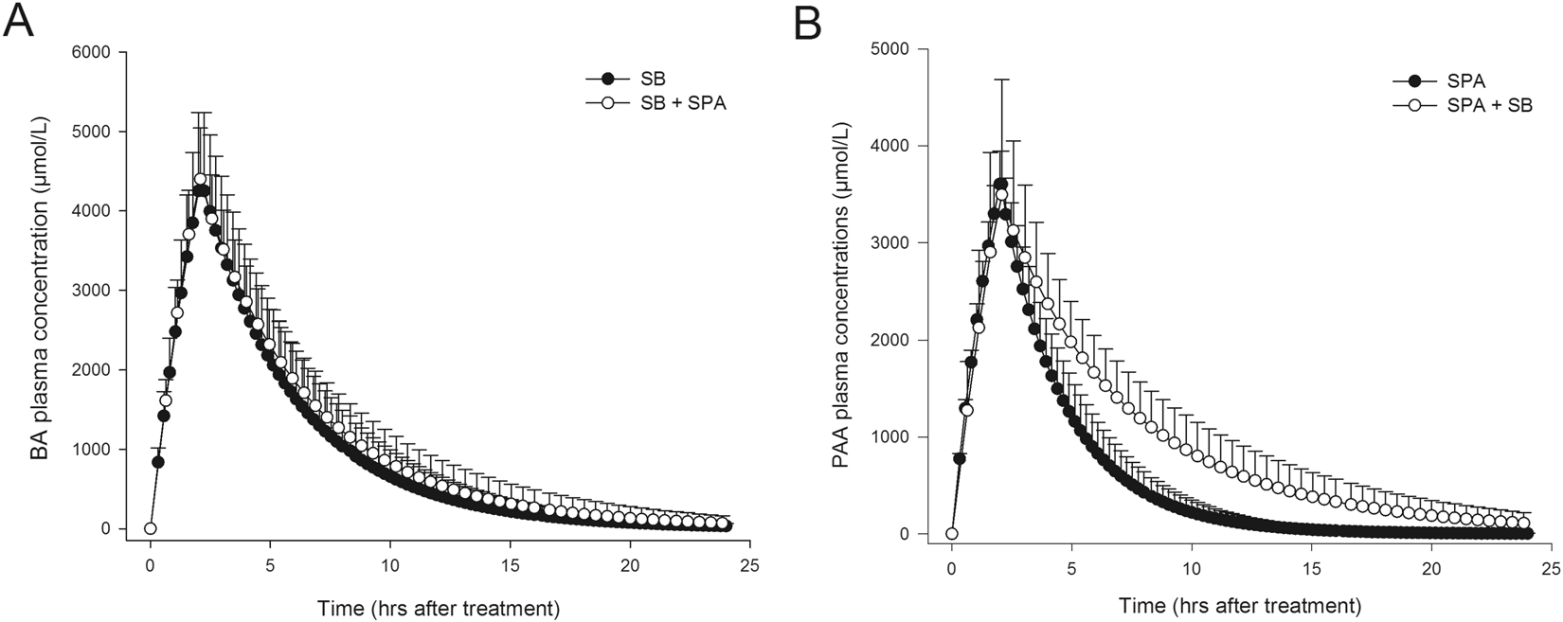
Simulation curves of active compounds in plasma, phase I. (**A**) Mean benzoic acid (BA) plasma concentrations following 250 mg/kg sodium benzoate (SB, closed dots) and SB + sodium phenylacetate (SPA, open dots) two hour-bolus administration in healthy dogs (n = 11). (**B**) Mean phenylacetic acid (PAA) plasma concentrations following 250 mg/kg SPA (closed dots) and SB + SPA (open dots) two hour-bolus administration in healthy dogs (n = 12).

Plasma pharmacokinetic parameters of the active substance of SB (benzoic acid, BA) and the active substance of SPA (phenylacetic acid, PAA) are summarized in Table 1. The large standard deviation demonstrates great inter-individual differences in the measured parameters, particularly during exposure to SB (Fig. 3A). Following SB and SPA as monotherapy, plasma levels of both BA and PAA had a maximum of 4265 µM and 3603 µM, respectively (Fig. 3). In addition, T½, AUC, and Vd values for BA were, 2.7 hours, 21.7 mM*h, and 0.756 L/kg for PAA, they were 2.4 hours, 15.0 mM*h and 0.797 L/kg (Table 1).

**Table 1:** Plasma pharmacokinetics of active compounds.

Analyte	Treatment	n	Cmax (µM)	T ½ (hours)	AUC (mM/h)	Vd (L/kg)
BA	SB	11	4,265 (941)	2.7 (0.5)	21.7 (6.1)	0.80 (0.23)
	SB + SPA	11	4,399 (643)	3.5 (1.3)	26.1 (8.3)	
PAA	SPA	10	3,603 (343)	2.4 (0.6)	15.0 (3.6)	0.76 (0.07)
	SB + SPA	12	3,494 (1,189)	4.2 (1.4)*	23.8 (6.7)*	

Pharmacokinetics of (analyte) benzoic acid (BA) and phenylacetic acid (PAA) during treatments with sodium benzoate (SB), sodium phenylacetate (SPA) and SB + SPA: n = number of dogs; Cmax, maximum plasma concentration; T½, half-life value; AUC, area under the concentration versus time curve; Vd, volume of distribution. Values are mean (SD). *Significant difference (P < 0.05) between monotherapy (SPA) and the combination therapy (SB + SPA).

Combined treatment (SB + SPA) significantly increased the T½ (P < 0.01) and AUC (P < 0.01) of PAA, when compared to the SPA alone. The increase in T½ of BA after combination treatment (SB + SPA) was not statistically significant (P = 0.06). After administration of NaCl 0.9%, levels of BA ware barely detectable (range 3–6 µmol/l) and not detectable for PAA (data not shown).

### Metabolites of SB and SPA in plasma

**Figure 4:**
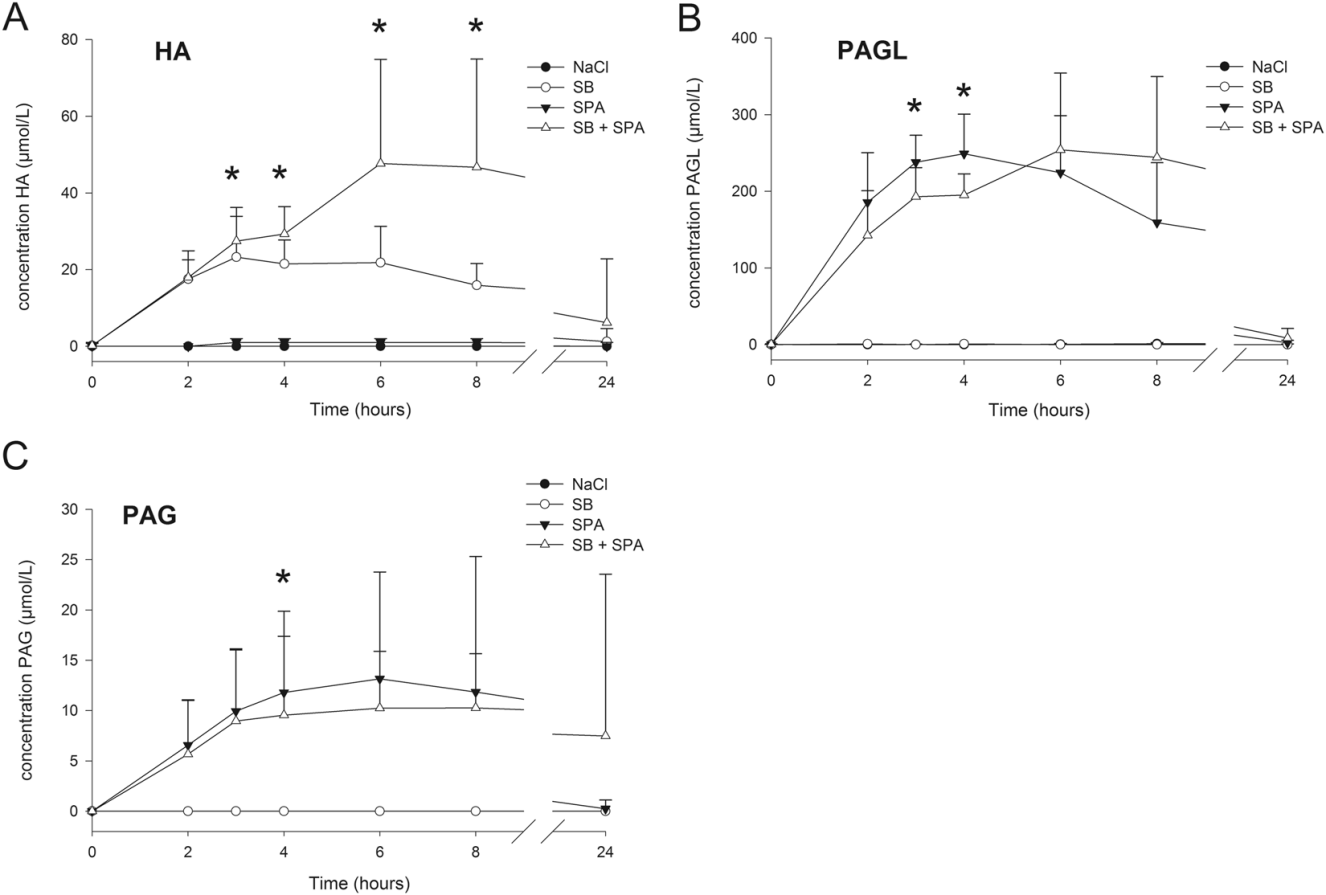
Concentration of SB and SPA metabolites in plasma of healthy dogs. (**A**) Plasma concentration of hippuric acid (HA), (**B**) phenylacetylglycine (PAGL), and (**C**) phenylacetylglutamine (PAG), after treatment with sodium benzoate (SB), sodium phenylacetate (SPA), the combination of SB + SPA, and NaCl (0.9%). T0, pre-treatment value; T2 = value after the end of the 2 hour bolus dose; T3-T24, times from the beginning of the treatment. *Significant difference (P < 0.05) between monotherapy (SB or SPA) and combination therapy (SB + SPA). Data is represented as mean + Standard Deviation (SD).

The administration of SB resulted in formation of HA with the highest plasma concentration (22 µmol/l), one and two hours after bolus infusion. Thereafter, concentrations gradually decreased and reached pre-treatment levels 24 hours post treatment (Fig. 4A, Supplementary Table S2). The combination of SB + SPA demonstrated significantly higher plasma HA concentration, from three to eight hours after treatment, when compared to SB alone. After 24 hours, HA concentrations returned to pre-treatment values (Fig. 4A, Supplementary Table S2). Following the bolus dose of SPA, plasma levels of both PAGL and PAG increased and reached maximum concentrations after four and six hours, respectively (Fig. 4B and C, Supplementary Table S2). Peak concentrations of PAGL (254 µmol/l) were more than 20-fold higher than those of PAG (11 µmol/l). After SB + SPA administration (and in comparison to SPA treatment alone), we observed significant lower levels of PAGL (T3–4) and PAG (T4) (Fig. 4B and C, Supplementary Table S2). At T24, both PAGL and PAG were almost undetectable. After administration of NaCl 0.9%, HA, PAGL and PAG were below detection level (Supplementary Table S2).

## Urinary excretion of SB and SPA metabolites and nitrogen in healthy dogs

Metabolite levels in urine resemble the levels found in plasma. Treatments resulted in significant increases in urine HA (SB, SB + SPA), PAGL and PAG (SPA, SB + SPA) levels. However, clear differences in excretion of these metabolites were observed when we compared each monotherapy treatment (SB or SPA) with the combination treatment (SB + SPA).

**Figure 5:**
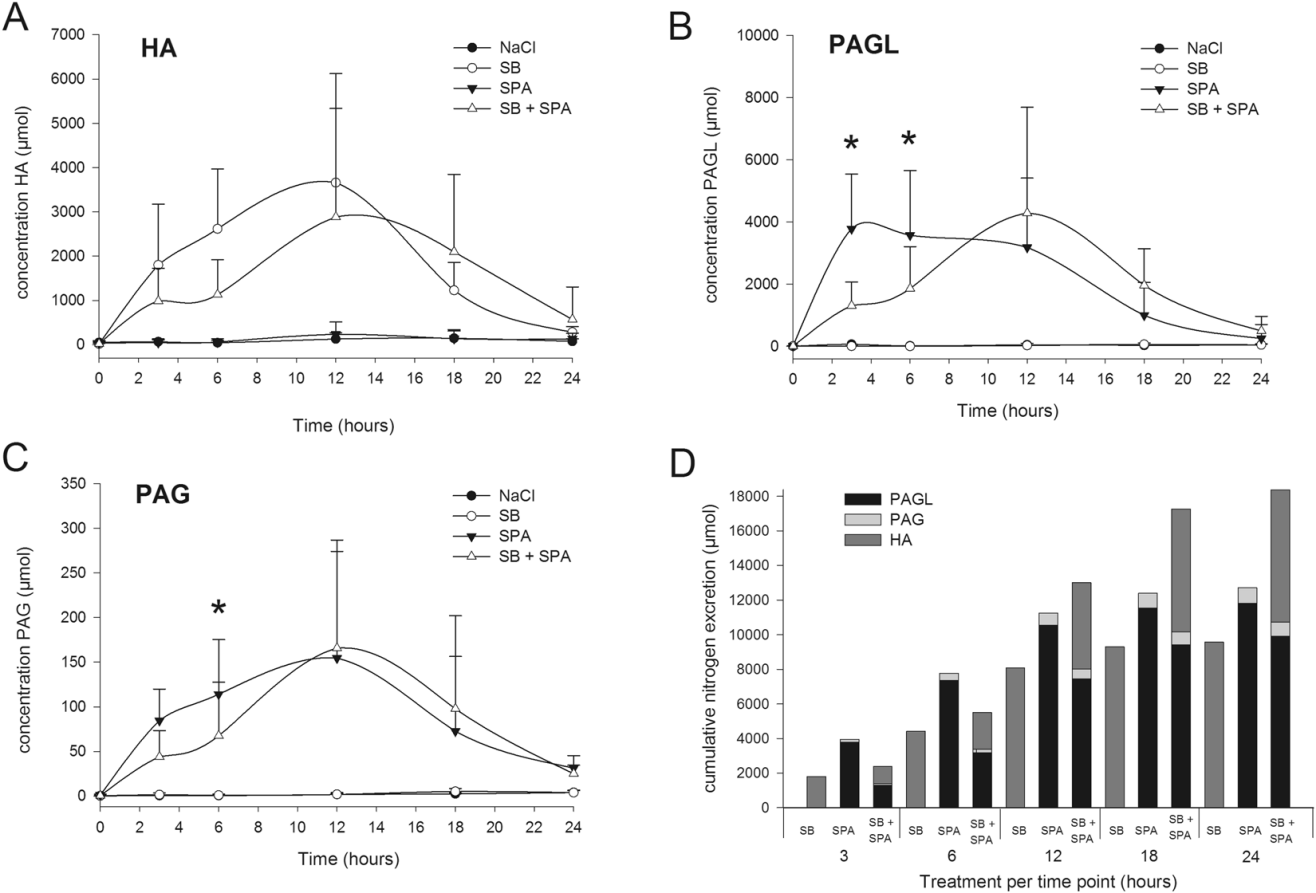
Excretion of SB and SPA metabolites and nitrogen in urine of healthy dogs. (**A**) Urinary (hippuric acid HA), (**B**) phenylacetylglycine (PAGL), and (**C**) phenylacetylglutamine (PAG), excretion (micromoles) after treatment with sodium benzoate (SB), sodium phenylacetate (SPA), 0.9% sodium chloride (NaCl), and the combination (SB + SPA). HA, PAG and PAGL are excreted faster after respectively SB and SPA treatments as mono-therapies compared to treatment with SB + SPA. (**D**) Estimated cumulative nitrogen excretion after treatments with SB (HA), SPA (PAGL + PAG), and SB + SPA (HA + PAGL + PAG). *Significant difference (P < 0.05) between mono therapy (SB or SPA) and the combination therapy (SB + SPA). Data is represented as mean + Standard Deviation (SD).

Treatment with SB + SPA during the first six hours resulted in significantly lower excretion rates of PAGL and PAG in comparison to treatment with SPA alone (Fig. 5B,C). A similar trend was observed with the excretion of HA after treatment with SB alone at T = 3 (P = 0.15) and T = 6 (P = 0.07) (Fig. 5A). Accordingly, the largest cumulative nitrogen excretion was also found to be at the first six hours after treatment with SPA as monotherapy. However, in the period between 12–24 hours after treatment, SB + SPA resulted in the highest nitrogen excretion rates (Fig. 5D). Urinary excretion of unchanged BA and PAA after SB and SPA administration respectively (Supplementary Table S3) was minimal (0.98% and 0.80% respectively). Unchanged BA and PAA after SB + SPA treatment was 2.24% and 2.16% respectively.

### Treatment effect on ammonia concentration in healthy dogs

In general, peak ammonia concentrations were measured at T2; however, we did not observe any significant differences in ammonia concentrations at T0, T2, T3 and T4 between the individual treatments, including NaCl 0.9% administration. Ammonia concentrations were all within the normal reference range. After NaCl 0.9% treatment ammonia concentrations were statistically significantly lower when compared with SB (T6, P = 0.02; T8, P = 0.04; T24, P = 0.03), SPA (T8, P = 0.02; T24, P = 0.01), and SB + SPA (T8, P = 0.04; T24, P = 0.04) (Supplementary Figure S1A).

### Safety, tolerability, pharmacokinetics and efficacy of SB administration in dogs with CPSS (phase II)

**Figure 6:**
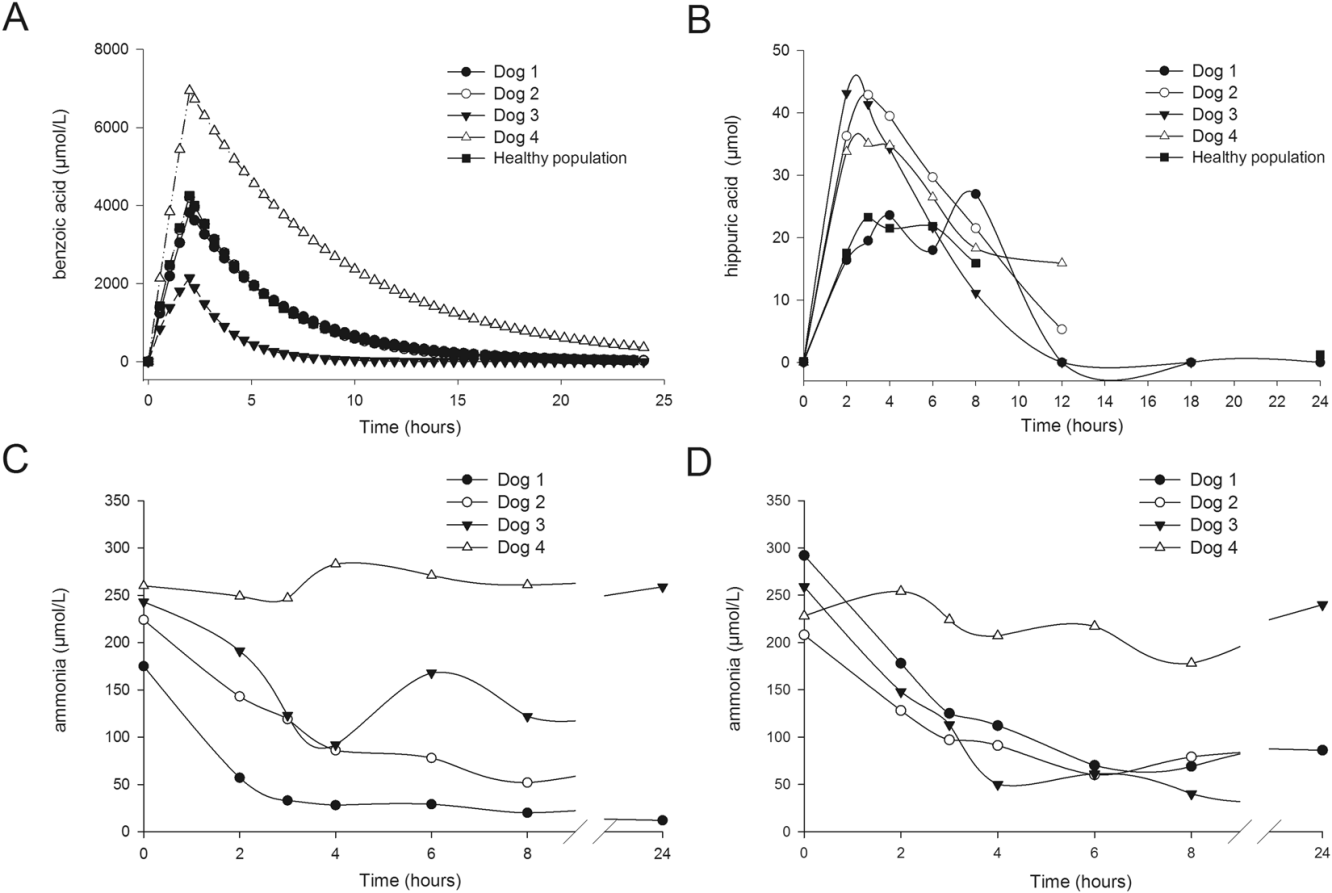
Simulation curve of active compound, metabolite concentrations and ammonia levels in shunt (CPSS) dogs. Plasma concentrations of (**A**) benzoic acid (BA) and (**B**) hippuric acid (HA) following a two hour-bolus administration of 250 mg/kg sodium benzoate (SB) in CPSS dogs (n = 4). Plasma concentration of ammonia after NaCl 0.9% (**C**) or SB administration (**D**) in CPSS dogs (n = 4). T0 indicates pre-treatment levels; T2 indicated directly after a bolus administration of SB or NaCl 0.9%; and T3-T24 indicates hours post treatment initiation (T3-T24).

Results obtained in phase I indicated that all drugs were well-tolerated. In addition, we observed that in dogs, SPA was primarily converted into PAGL as opposed to PAG in humans. Therefore, in regard to using the dog as a model for human hyperammonemia, we chose to only use SB in our CPSS dog population. In all dogs, no adverse effects were observed during or after treatment. Abnormal parameters seen in complete blood count (CBC) and biochemical blood analysis prior to treatment were typical for dogs with CPSS^36^ and included hyperammonemia (n = 4), mild anemia (n = 2), increased liver enzymes and bile acids (n = 4), hypoalbuminemia (n = 4), and leukocytosis (n = 4). During and after treatments, levels of electrolytes, glucose, and pH remained within reference values (Supplementary Figure S1B). Plasma pharmacokinetic parameters of BA after SB treatment are shown in Fig. 6A. Mean (SD) Cmax, T1/2 and AUC were 4,356 (1,976) µM, 3.3 (1.7) hours and 28,224 (24,245) h*µM respectively. Also, dogs with CPSS displayed great inter-individual differences within the measured parameters. No significant differences were found in the pharmacokinetic parameters between the healthy dog population (phase I) and dogs with CPSS (phase II). We observed that SB treatment in both populations resulted in increasing concentrations of HA that reached a maximum level between three and four hours post treatment initiation (Fig. 6B). HA was not detectable after NaCl administration (data not shown).

### Urinary excretion of HA in CPSS dogs

Urinary excretion of HA increased in all dogs after SB treatment and reached a maximum six hours after treatment initiation (Supplementary Figure S2).

### Treatment effect on ammonia concentration in CPSS dogs

Pre-treatment ammonia concentrations (180–260 µmol/l) were increased compared to reference values (15–45 µmol/l) in all dogs (Fig. 6C,D). Mean ammonia concentrations at T2, T3, T4, T6 and T8 were significantly reduced after both SB and NaCl treatments (in both treatments), although no significant differences in ammonia reductions were found between SB and NaCl treatments. In general, both SB and NaCl treatments reduced ammonia concentrations by approximately 35% at T2, and further by approximately 60% at T8 of the pre-treatment values. Despite the significant reduction in mean ammonia concentrations, a great variation was observed between the four dogs (Fig. 6C,D). In Dog 1, 2 and 3, both SB and NaCl treatments resulted in a decrease of ammonia within the first eight hours. In Dog 4, ammonia concentrations were refractory to both treatments, and remained high (>200 µmol/l) during the course of both treatments. In the first six hours after treatment, ammonia concentrations even increased after SB (T2) and NaCl (T4) treatments.

## Discussion

We assessed the potential of SB and SPA to reduce ammonia concentrations in a canine model of hyperammonemia. To our knowledge, this is the first randomized, placebo controlled study for the safety and efficacy of SB and SPA treatments of hyperammonemia. Due to the similarities of ammonia metabolism, dogs are considered a valid model for hyperammonemia in man^29,31^. In our study, SB and SPA treatments were safe and well-tolerated in both healthy dogs (SB + SPA) and dogs with CPSS (SB).

In general, the pharmacokinetics of BA and PAA in the healthy dog population and in the CPSS dogs was similar to those reported in human studies. Cmax, AUC and T½ values after SB + SPA treatment were comparable with values reported by Brusilow^7^ and MacArthur^37^. Green *et al*.^37^, administered SB as a monotherapy in newborn infants, and reported a mean T½ of BA of 2.8 hours, which is similar to the T½ found in our study (2.7 hours). Metabolism of BA in both the healthy and CPSS dog populations was comparable to human metabolism^9^. After SB treatment, BA was rapidly converted to HA which was excreted in the urine. These comparable finding in CPSS dogs are most likely the result of sufficient renal and/or hepatic metabolism of these substances. In CPSS dogs renal function is not reduced and hepatic arterial flow is increased as a compensatory mechanism to the decreased portal flow^38^. Metabolism of PAA, on the other hand, differed from that shown in humans. In humans, PAA is metabolized to PAG, while PAA in the studied dogs was metabolized mainly to PAGL (95%) and only to a small extent, PAG (5%). This is in contrast to previous studies^12,13^ reporting that PAA can be metabolized to PAG in humans and higher primates but not in dogs. To our knowledge, this is the first study showing formation of both PAGL and PAG from PAA in the dog. The preference of PAGL formation may be explained by the fact that both glutamine- and glycine-N phenylacetyltransferase are present in the dog, with the latter having a higher activity, consequently favoring PAGL formation over that of PAG. Therefore, we recommend using CPSS dogs as a naturally occurring model for treatment of human hyperammonemia with SB but not with SPA.

The objective of SB and SPA therapy in the treatment of acute hyperammonemia is to form and excrete as much HA, PAGL and PAG (i.e. nitrogen) as possible within the shortest time in order to decrease and preferably normalize plasma ammonia levels. In our present study, we observed clear differences in the formation and excretion of HA, PAGL and PAG during treatments. Treatment with SPA alone resulted in the highest cumulative urinary nitrogen excretion within the first six hours after treatment while SB + SPA treatment has resulted in the highest nitrogen excretion from 12 to 24 hours after treatment. Previous studies^39,40^ have shown that the substrates of HA and PAGL/PAG formation compete with each other, and consequently result in decreased production of these compounds. More specifically, HA and PAGL in dogs, are virtually the only end products of BA and PAA, respectively; thus both phenylacetyl-CoA and benzoyl-CoA compete for the same glycine molecule. Competition also occurs between the metabolites in the same tubular transport system in the kidney. Together, these may explain the reduction in urinary excretion of HA, PAG and PAGL after the combination treatment with SB + SPA.

As in human studies, treatment with SB and SPA resulted in increased excretion of nitrogen containing compounds (i.e. HA, PAG and PAGL). However, efficacy of a treatment for hyperammonemia should not be evaluated on the excretion of nitrogen containing compounds alone but rather on its effect on ammonia levels. Therefore, in the current study, the efficacy of SB and SPA was assessed based on reduction of ammonia concentrations after treatments compared to a placebo treatment with NaCl 0.9%. The most striking observation of our study was that treatment of hyperammonemia with SB was not better than placebo; we observed similar reductions in ammonia concentrations in the healthy phase I dogs. We speculate that mechanisms other than excretion of surplus nitrogen via alternative pathways may be responsible for the relatively rapid decrease in ammonia concentration after intravenous bolus treatment with SB and SPA. One such mechanism is volume expansion and consequently forced diuresis, as the only factor common to verum and placebo treatments was the high fluid volume (12.5 ml/kg^/^h) administered during the two-hour bolus treatments. In current UCED patient treatment regimens, the effect of volume expansion is even fortified with the use of hypertonic dextrose 10% solution (15 ml/kg/h) during the two-hour bolus injection of SB + SPA^1,7^. For both humans and dogs, these volumes are approximately three to five times higher than the recommended maintenance fluid requirements per hour^41–44^, thus eventually resulting in volume expansion and stimulation of forced diuresis. This is consistent with a study performed in patients with hyperammonemia due to liver cirrhosis^45^. Volume expansion (administration of one liter of NaCl 0.9% in one hour) resulted in a 40% reduction of ammonia concentration by significantly increasing ammonia excretion and reducing angiotensin II (ANG II) production. The latter effect is important because ANG II enhances ammoniagenesis in the proximal tubules^46^. Improved systemic perfusion, as a result of volume expansion, also increases uptake of ammonia by skeletal muscle and the brain where it can be detoxified^44^.

In this study, ammonia levels in one CPSS dog (Dog 4) were refractory to both SB and NaCl 0.9% treatments. Interestingly, metabolism of BA and excretion of HA in this patient were comparable to that of the other three dogs indicating adequate production of metabolites. Since dog 4 was presented with vomiting and anorexia prior to referral a possible explanation for the lack of response in this dog could be the presence of a catabolic state. A catabolic state increases ammonia production due to protein degradation thereby limiting the effect of increased excretion of ammonia by forced diuresis. This lack of response to treatment was also previously reported in some UCED patients treated with SB and SPA^7,8,10,11^. These patients most likely require more aggressive treatment methods, such as exchange transfusions, to reduce ammonia concentrations.

In conclusion, tolerability, safety, metabolism and pharmacokinetics of SB and SPA in CPSS dogs are comparable to healthy dogs and humans (with the exception of PAGL being the end product of PAA in dogs rather than PAG in humans). The results of the current study (although in a limited number of dogs) suggest that dogs with CPSS are an excellent model to study treatment of hyperammonemia with SB. However, saline is as effective as nitrogen scavengers for treatment of hyperammonemia. This suggests effects of volume expansion and/or forced diuresis rather than increased production of nitrogenous waste. Consequentially, treatment of hyperammonemia justifies additional/placebo-controlled trials in human medicine. Extending the standard treatment in human patients with additional forced diuresis treatment as results from high volume isotonic crystalloid administration might potentially fortify the ammonium lowering effect.

## Electronic supplementary material

Supplementary files
