# Supplementary material for: Saline is as effective as nitrogen scavengers for treatment of hyperammonemia
Source: Sci Rep. 2017 Oct 13;7:13112. doi: 10.1038/s41598-017-12686-9 (PMC5640627; doi:10.1038/s41598-017-12686-9)
Supplement: Supplementary file 1 — Supplementary files [file 41598_2017_12686_MOESM1_ESM.pdf]

Manuscript title: Saline is as effective as nitrogen scavengers for treatment of hyperammonemia

G van Straten[a,*], M G M de Sain-van der Velden[b], I M van Geijlswijk[c], R P Favier[a], S J Mesu[c], N E Holwerda-Loof[b,†], M van der Ham[b], H Fieten[a], J Rothuizen[a], B Spee[a,1] and N M Verhoeven-Duif[b,1]

[a]Department of Clinical Sciences of Companion Animals, Faculty of Veterinary Medicine, Utrecht University, Utrecht, The Netherlands.

[b]Department of Medical Genetics, Wilhelmina Children's Hospital, University Medical Centre (UMC) Utrecht, The Netherlands.

[c]Pharmacy Department, Faculty of Veterinary Medicine, Utrecht University, Utrecht, The Netherlands.

[*] ***Corresponding author****:* G. van Straten, DVM, PhD, Department of Clinical Sciences of Companion Animals, Faculty of Veterinary Medicine, Utrecht University. Yalelaan 8, 3508 TD Utrecht PO Box: 80154, The Netherlands. E-mail: G.vanStraten@uu.nl.
[1]equally contributed to this work

[†]Deceased January 2016. This article is dedicated to her memory.

Supplementary Materials

Table S1. Blood analysis, phase I (A) and phase II (B). Mean concentration (n=12) of Potassium (K, mmol/L), Calcium (Ca, mmol/L), Albumin (Alb, g/L), blood pH, urea (BUN, mmol/L), Creatinine (Creat, µmol/L), Alkaline phosphatase (AP, IU/L), Alanine-aminotransferase (ALAT, IU/L), Bile acids (µmol/L), Total protein (TP, g/L), and Hematocrit (Ht, L/L), before and after treatment with 0.9% sodium chloride (NaCl), sodium benzoate (SB), sodium phenylacetate (SPA), or combination therapy (SB+SPA).

A.

Treatment	Time	Na		K		Ca		Alb		pH		BUN		Creat		AP		ALAT		Bile acids		TP		Ht	
		Mean	SD	Mean	SD	Mean	SD	Mean	SD	Mean	SD	Mean	SD	Mean	SD	Mean	SD	Mean	SD	Mean	SD	Mean	SD	Mean	SD
NaCl	0	149.3	2.0	4.16	0.32	2.51	0.08	28.8	1.4	7.40	0.03	8.9	2.0	72	13	45	30	79	52	16.3	16.6	57.4	3.9	0.45	0.02
NaCl	2	148.9	1.7	3.76	0.27	2.43	0.08	26.9	1.7	7.40	0.03	--	--	--	--	--	--	--	--	--	--	--	--	--	--
NaCl	3	148.5	2.4	3.78	0.28	2.48	0.06	27.5	1.1	7.41	0.02	--	--	--	--	--	--	--	--	--	--	--	--	--	--
NaCl	4	148.6	2.4	3.87	0.40	2.47	0.05	27.8	1.1	7.41	0.02	--	--	--	--	--	--	--	--	--	--	--	--	--	--
NaCl	6	148.8	1.1	3.77	0.28	2.51	0.05	28.8	1.1	7.42	0.02	--	--	--	--	--	--	--	--	--	--	--	--	--	--
NaCl	8	148.5	1.2	3.85	0.20	2.52	0.05	29.4	1.3	7.41	0.02	--	--	--	--	--	--	--	--	--	--	--	--	--	--
NaCl	24	148.1	1.3	3.88	0.34	2.53	0.06	29.7	1.6	7.42	0.02	3.9	1.1	64	15	53	30	76	41	3.2	1.6	59.0	2.4	0.43	0.02
SB	0	149.0	1.8	4.27	0.30	2.52	0.07	28.0	1.9	7.41	0.02	7.9	1.5	71	11	102	191	518	1267	35.9	36.8	58.1	3.4	0.45	0.06
SB	2	146.1	1.6	4.15	0.14	2.49	0.08	27.2	2.2	7.44	0.03	--	--	--	--	--	--	--	--	--	--	--	--	--	--
SB	3	146.5	1.9	3.89	0.17	2.50	0.05	27.8	2.1	7.45	0.02	--	--	--	--	--	--	--	--	--	--	--	--	--	--
SB	4	146.5	1.8	3.84	0.16	2.50	0.05	27.4	2.1	7.46	0.02	--	--	--	--	--	--	--	--	--	--	--	--	--	--
SB	6	146.8	2.2	3.91	0.26	2.51	0.04	27.8	2.1	7.45	0.02	--	--	--	--	--	--	--	--	--	--	--	--	--	--
SB	8	148.1	1.1	3.87	0.19	2.55	0.05	28.1	2.0	7.45	0.02	--	--	--	--	--	--	--	--	--	--	--	--	--	--
SB	24	147.9	1.8	3.89	0.18	2.58	0.05	29.2	1.7	7.43	0.01	5.2	1.7	66	12	99	177	450	1004	4.3	3.5	57.8	2.4	0.43	0.05
SPA	0	147.8	3.4	4.23	0.19	2.55	0.06	27.0	3.7	7.40	0.03	8.4	1.8	74	12	125	274	457	1311	24.4	17.9	59.4	5.9	0.42	0.04
SPA	2	145.4	2.6	3.81	0.21	2.51	0.09	27.2	3.7	7.45	0.02	--	--	--	--	--	--	--	--	--	--	--	--	--	--
SPA	3	144.8	2.5	3.79	0.23	2.53	0.06	27.4	3.4	7.47	0.03	--	--	--	--	--	--	--	--	--	--	--	--	--	--
SPA	4	145.2	2.6	3.75	0.19	2.54	0.07	27.5	3.4	7.46	0.02	--	--	--	--	--	--	--	--	--	--	--	--	--	--
SPA	6	147.3	3.1	3.94	0.17	2.55	0.06	28.1	3.5	7.46	0.03	--	--	--	--	--	--	--	--	--	--	--	--	--	--
SPA	8	147.6	2.6	3.90	0.26	2.56	0.06	28.1	3.6	7.44	0.03	--	--	--	--	--	--	--	--	--	--	--	--	--	--
SPA	24	147.2	3.1	3.82	0.21	2.61	0.05	29.1	3.7	7.42	0.03	5.2	1.2	70	11	124	258	445	1234	17.0	37.2	60.6	5.1	0.42	0.03
SB+SPA	0	149.0	2.2	4.33	0.22	2.49	0.07	28.3	1.7	7.40	0.02	7.4	2.2	71	11	49	24	143	92	26.8	25.0	59.1	4.2	0.42	0.02
SB+SPA	2	147.4	2.6	3.97	0.35	2.45	0.09	27.8	2.6	7.45	0.02	--	--	--	--	--	--	--	--	--	--	--	--	--	--
SB+SPA	3	147.1	2.2	3.94	0.22	2.47	0.07	27.9	2.3	7.49	0.10	--	--	--	--	--	--	--	--	--	--	--	--	--	--
SB+SPA	4	147.1	2.3	3.92	0.25	2.50	0.09	28.5	2.7	7.46	0.02	--	--	--	--	--	--	--	--	--	--	--	--	--	--
SB+SPA	6	146.9	1.8	3.93	0.17	2.49	0.09	28.0	2.5	7.46	0.03	--	--	--	--	--	--	--	--	--	--	--	--	--	--
SB+SPA	8	146.8	1.7	3.83	0.19	2.49	0.09	27.7	2.3	7.45	0.03	--	--	--	--	--	--	--	--	--	--	--	--	--	--
SB+SPA	24	146.2	3.6	3.93	0.31	2.56	0.10	28.6	3.6	7.42	0.04	12.8	15.4	119	89	108	148	155	103	3.3	1.9	60.9	5.3	0.42	0.04

B.

Treatment	Time	glucose		Na		K		pH	
		Mean	SD	Mean	SD	Mean	SD	Mean	SD
**NaCl**	0	6.3	1.1	144	4.2	3.8	0.24	7.46	0.03
**NaCl**	2	6.8	1.7	145	3.6	3.9	0.38	7.41	0.02
**NaCl**	2	6.7	1.1	146	3.9	4.0	0.38	7.43	0.02
**NaCl**	4	6.0	0.5	145	5.5	4.0	0.42	7.42	0.02
**NaCl**	6	5.2	0.7	144	1.7	3.9	0.36	7.43	0.04
**NaCl**	8	5.4	0.7	146	4.3	4.3	0.84	7.42	0.02
**NaCl**	24	5.0	0.8	147	1.2	4.1	0.45	7.43	0.03
**SB**	0	5.6	1.0	145	2.1	4.0	0.61	7.38	0.05
**SB**	2	6.1	0.5	143	3.0	3.7	0.20	7.39	0.05
**SB**	3	5.9	0.8	142	1.7	4.0	0.50	7.40	0.03
**SB**	4	5.7	1.0	142	1.0	3.7	0.05	7.44	0.03
**SB**	6	5.4	0.5	145	3.4	4.0	0.34	7.43	0.01
**SB**	8	5.5	0.6	143	3.2	3.9	0.17	7.42	0.05
**SB**	24	5.0	0.1	144	4.0	3.8	0.30	7.42	0.04

Table S2. Concentration of metabolites in plasma of healthy dogs. Median plasma concentrations (range) of hippuric acid (HA), phenylacetylglycine (PAGL) and phenylacetylglutamine (PAG) after treatment with sodium benzoate (SB), sodium phenylacetate (SPA), the combination of SB+ SPA, and NaCl (0.9%). T0= pre-treatment value. T2= value after the end of the 2 hour bolus dose. T3-T24 = hours post treatment. ND=not detectable (<1 μmol/L). *= significant difference (*P*<0.05) between mono therapy (SB or SPA) and the combination therapy (SB+SPA).

Analyte	Treatment	T0	T2	T3	T4	T6	T8	T24
HA (µmol/L)	SB	ND	18 (7-25)	22 (9-50)	22 (9-30)	19 (9-45)	16 (5-25)	1 (1-11)
	SB+SPA	ND	18 (1-26)	28 (17-47)*	29 (18-40)*	37 (24-102)*	33 (22-101)*	2 (1-372)
	NaCl	ND	ND	ND	ND	ND	ND	ND
PAGL (µmol/L)	SPA	1 (1-2)	193 (1-250)	240 (167-296)	254 (149-330)	247 (54-323)	170 (8-286)	2 (1-11)
	SB+SPA	1 (0-2)	155 (1-231)	191 (140-275)*	197 (152-244)*	224 (147-447)	201 (133-439)	7 (1-1022)
	NaCl	ND	ND	ND	ND	ND	ND	ND
PAG (µmol/L)	SPA	ND	6 (1-19)	9 (5-28)	10 (6-37)	11 (4-46)	9 (1-53)	1 (1-3)
	SB+SPA	ND	4 (0-22)	7 (4-31)	7 (5-34)*	8 (4-27)	9 (5-26)	1 (1-43)
	NaCl	ND	ND	ND	ND	ND	ND	ND

Table S3. Concentration of metabolites in urine of healthy dogs. Excretion of HA, PAGL and PAG after treatment with SB, SPA and SB+SPA. Values are median (range). T3-T24= times after beginning of the treatment. Recovery is the amount of excreted HA or PAGL and PAG divided by the administrated amount of respectively SB or SPA provided in percentages. BA and PAA urine excretion are given as percentages of the administrated moles of respectively SB and SPA.

Analyte	Treatment	Total administrated SB* or SPA** (μmol)	T3	T6	T12	T18	T24	Total excreted (μmol)	Percentage recovery of BA as HA	Percentage recovery of PAA as PAGL or PAG	Percentage unchanged BA	Percentage unchanged PAA
HA	SB*	18,874	1,492 (620-4,558)	2,741 (958-4,523)	2,705 (1,119-6,944)	1,535 (62-1,711)	212 (136-497)	8,685	39 (19-62)		0.98	
	SB+SPA*	20,262	538 (101-2,101)	989 (138-2,532)	2,013 (201-6,267)	1,847 (329-6,359)	346 (101-2,101	5,733	27 (8-58)		2.24	
	NaCl		15 (2-229)	14 (1-132)	27 (1-336)	40 (1-434)	105 (9-114)	201				
PAGL	SPA**	19,446	4,192 (1,131-6,357)	3,545 (671-6,963)	3,150 (846-7,175)	486 (93-3,215)	63 (10-1,430)	11,436		60 (33-93)		0.80
	SB+SPA**	18,466	1,095 (538-3,797)	2,128 (305-4,333)	4,026 (491-10,221)	1,362 (894-4,381)	401 (69-1,369)	9,012		46 (20-76)		2.16
	NaCl		15 (2-229)	12 (3-37)	29 (2-155)	28 (2-71)	59 (24-67)	143				
PAG	SPA**	19,446	78 (34-127)	130 (15-218)	125 (30-507)	26 (3-466)	6 (1-199)	364		2 (1-7)		
	SB+SPA**	18,466	37 (9-88)	65 (8-193)	191 (13-300)	89 (37-213)	22 (5-64)	404		2 (1-4)		
	NaCl		1 (1-1)	1 (1-1)	1 (1-1)	3 (1-4)	5 (2-6)	11				

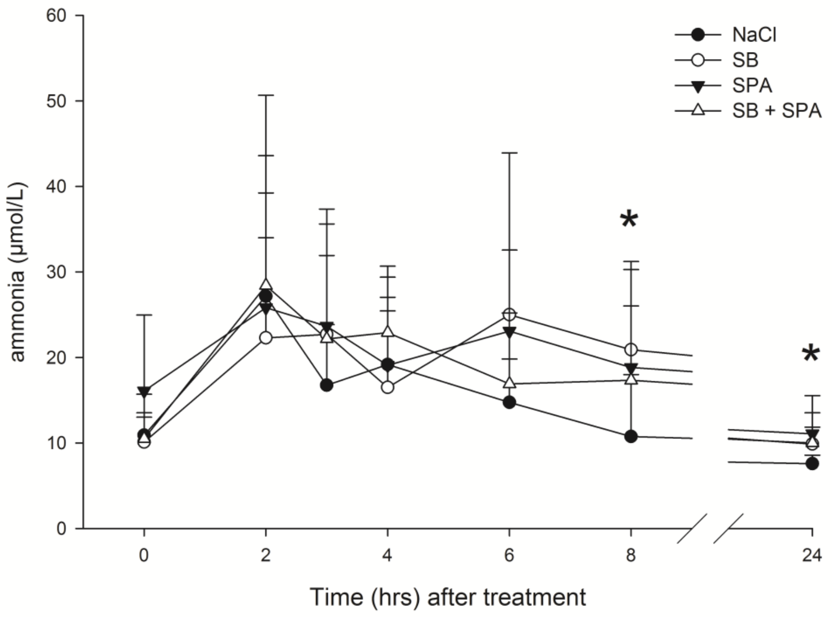

Fig. S1. Blood ammonia levels in healthy dogs after treatment. Ammonia concentrations prior to treatment (T0) and after administration of NaCl (0.9%), SB, SPA and SB+SPA (T2-T24) in healthy dogs (n=12). *= significant difference (*P*<0.05) between NaCl and mono (SB or SPA) or combination (SB+SPA) therapies.

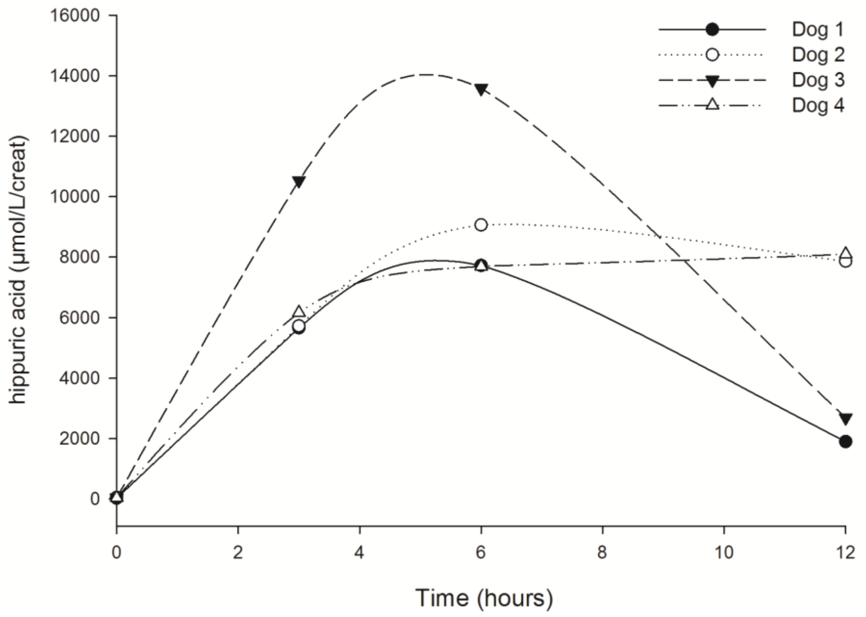

Fig. S2. Concentration of metabolite HA in urine of shunt dogs. HA concentrations in urine (µM/mmol creatinine) after treatment with SB in CPSS dogs (n=4).
